# Factors, associated with elevated concentration of soluble carbonic anhydrase IX in plasma of women with cervical dysplasia

**DOI:** 10.1038/s41598-022-19492-y

**Published:** 2022-09-13

**Authors:** Švitrigailė Grincevičienė, Daiva Vaitkienė, Daiva Kanopienė, Rasa Vansevičiūtė, Jan Tykvart, Artūras Sukovas, Joana Celiešiūtė, Ernesta Ivanauskaitė Didžiokienė, Arvydas Čižauskas, Aida Laurinavičienė, Vlastimil Král, Anna Hlavačková, Jitka Zemanová, Dovilė Stravinskienė, Aistė Sližienė, Agnė Petrošiūtė, Vytautas Petrauskas, Renata Balsytė, Jonas Grincevičius, Vaclav Navratil, Ullrich Jahn, Jan Konvalinka, Aurelija Žvirblienė, Daumantas Matulis, Jurgita Matulienė

**Affiliations:** 1 Department of Biothermodynamics and Drug Design, Institute of Biotechnology, Life Sciences Center, Vilnius University, Sauletekio sl. 7, 10257 Vilnius, Lithuania; 2 Department of Obstetrics and Gynecology, Medical Academy, Lithuanian University of Health Sciences, Eiveniu st. 2, 50161 Kaunas, Lithuania; 3 Consultative Polyclinic Department, National Cancer Institute, Santariskiu st. 1, 08406 Vilnius, Lithuania; 4 Clinic of Obstetrics and Gynecology, Faculty of Medicine, Institute of Clinical Medicine, Vilnius University, M. K. Ciurlionio st. 21, 03101 Vilnius, Lithuania; 5 Diana Biotechnologies, Nad Safinou II 366, 252 50 Vestec, Czech Republic; 6 National Center of Pathology, Affiliate of Vilnius University Hospital Santaros Klinikos, P. Baublio st. 5, 08406 Vilnius, Lithuania; 7 Department of Pathological Anatomy, Medical Academy, Lithuanian University of Health Sciences, Eiveniu st. 2, 50161 Kaunas, Lithuania; 8 Department of Pathology, Forensic Medicine and Pharmacology, Institute of Biomedical Science, Faculty of Medicine, Vilnius University, M. K. Ciurlionio st. 21, 03101 Vilnius, Lithuania; 9 Institute of Molecular Genetics of the Czech Academy of Sciences, Flemingovo n. 2, 166 37 Prague 6, Czech Republic; 10 Institute of Organic Chemistry and Biochemistry of the Czech Academy of Sciences, Flemingovo náměstí 542/2, 160 00 Praha, Czech Republic; 11 Department of Immunology and Cell Biology, Institute of Biotechnology, Life Sciences Center, Vilnius University, Sauletekio sl. 7, 10257 Vilnius, Lithuania; 12 Pharmacy Center, Institute of Biomedical Science, Faculty of Medicine, Vilnius University, M. K. Ciurlionio st. 21, 03101 Vilnius, Lithuania

**Keywords:** Diseases, Risk factors

## Abstract

Precancerous lesions of human cervix uteri have a tendency for regression or progression. In cervical intraepithelial neoplasia grade 2 (CINII) case there is an uncertainty if a lesion will progress or regress. The carbonic anhydrase IX (CAIX) enzyme is overexpressed in cervical cancer which is more sensitive to radiotherapy. CAIX is associated with poor prognosis in solid hypoxic tumors. The aim of this study was to determine factors related to elevated soluble CAIX (s-CAIX) in high-grade intraepithelial lesion (HSIL) cases. Methods. Patients diagnosed with HSIL (N = 77) were included into the research group whereas without HSIL (N = 72)—the control group. Concentration of the soluble CAIX (s-CAIX) in plasma was determined by the DIANA ligand-antibody-based method. *C. trachomatis* was detected from cervical samples by PCR. Primary outcomes were risk factors elevating s-CAIX level in HSIL group. Non-parametric statistical analysis methods were used to calculate correlations. Results. The s-CAIX level in patients with HSIL was elevated among older participants (r_s_ = 0.27, *p* = 0.04) and with *C. trachomatis* infection (*p* = 0.028). Among heavy smokers with HSIL, the concentration of s-CAIX was higher in older women (r_s_ = 0.52, *p* = 0.005), but was not related to the age of heavy smokers’ controls (τ = 0.18 *p* = 0.40). Conclusion. The concentration of s-CAIX was higher among older, heavy smoking and diagnosed with *C. trachomatis* patients. All these factors increased the risk for HSIL progression.

## Introduction

Since cervical cancer affects many women in the World^1^, different effective strategies have been developed to reduce both the disease incidence rate and mortality, such as cervical cytology and high-risk human papillomavirus (hr-HPV) detection as well as vaccination against most prevalent virus types^2^. However, there is still a long path to disease elimination^3^.

Cervical cytology and co-testing hr-HPV for triage helps to identify moderate or severe dysplasia and carcinoma in situ^4,5^. Conization is a golden standard treatment for cervical cancer prevention, but increases risk of adverse events for fertility and pregnancy^6–8^, such as miscarriage or preterm birth^7,8^. In the contexts of eldering Western society, due to later parenthood planning, it is better to avoid unnecessary interventions^9^.

Precancerous lesions tend to regress among younger patients^10^ and persist or progress for older ones^11–14^. The observatory strategy increases the patient anxiety, healthcare cost and the number of office visits^15,16^. Moreover, the testing has a substantial false negative rate^17,18^. Women after 30 may have higher probability of atypical squamous cells of undetermined significance (ASCUS), low-grade intraepithelial lesion (LSIL) with false negative hr-HPV of 12.9%^19^, and a higher probability to miss high-grade intraepithelial lesion HSIL cases^5,20^. Cervical intraepithelial neoplasia grade 2 (CINII) false negative hr-HPV makes 6.2%^19^. Therefore, it is necessary for individualized markers helping to predict and stratify patients who could still chose an observatory strategy and who should be treated more aggressively.

One of such markers could be carbonic anhydrase IX (CAIX). There are 15 carbonic anhydrase isoforms in human body (numbered from I to XIV because there are two isoforms of CAV, CAVA and CAVB)^21–23^. Five isoforms are expressed in the cell cytoplasm (CAI, CAII, CAIII, CAVII and CAXIII), two, CAVA and CAVB – in the mitochondria, the CAVI is excreted, three—CAVIII, CAX and CAXI—are catalytically inactive isoforms, and the remaining four—CAIV, CAIX, CAXII and CAXIV—are expressed extracellularly attached to cell membrane^21–23^. Carbonic anhydrase is important for pH regulation because it catalyzes CO_2_ hydration into bicarbonate and acid protons^22^.

The isoform IX (CAIX) has been detected in human neoplasms, such as solid hypoxic cancers^24–26^ and also in precancerous lesions of cervix and cervical cancer^27,28^. Carbohydrase IX soluble isoform (s-CAIX) and expression in tissue was detected in human plasma among patients with oncogynecological diseases^29–31^. In breast cancer patients s-CAIX detection helped to identify a patient group who would not benefit from bevacizumab treatment^32^. Together with other markers s-CAIX level was a prognostic marker for renal cell carcinoma^33^. The protein was measured in urine and was elevated six months prior transitional cell carcinoma diagnosis^34^. In non-small cell lung cancer and vulvar cancer patients s-CAIX was independent poor overall survival prediction biomarker^35,36^.

The objectives of this study were following: (1) detect s-CAIX concentration in blood plasma in the ≥HSIL and <HSIL groups and (2) to compare s-CAIX level among the women with risk factor for HSIL progression, such as age, sexually transmitted disease *C. trachomatis*, smoking status, and contraception. Our hypothesis was that s-CAIX level should be associated with risk factors for disease progression contributing to systemic inflammation in carcinogenesis^37^. We conducted a cross-sectional study with 2 groups: research group participants had histologically confirmed ≥HSIL and control group without high grade intracervical dysplasia.

## Materials and methods

### Study participants

Adult patients (23–65 years) who were referred for triennial Papanicolaou (PAP) smear (National cervical cancer screening program) to Onos Gurevičienės Family Clinic in Marijampole city and received negative result—negative for intraepithelial lesion malignancy (NILM) were prospectively enrolled for control group from 15th of March 2016 to 30th of December 2018. Women of the same age who were referred for conization because of suspected high-grade intracervical neoplasia (HSIL) on PAP smear or biopsies to main university affiliated tertial oncogynecology centers (National Cancer Institution and Kauno Klinikos, Lithuanian University of Health Sciences) were prospectively enrolled into the research group during the same period. Exclusion criteria were the pre-existing oncological pathology, endometrial hyperplasia, or human immunodeficiency virus. Respondents filled questionnaire about their age, sexual and reproductive history and contraception choice. Sexual history, especially risky sexual behavior is associated with higher probability of hr-HPV acquisition^38^. Women were asked about their smoking status because scientific literature provides evidence of its relation to HSIL pathology and oncogenic process^38^. Women were tested for *C. trachomatis*, that is also known as a co-factor in cervical cancerogenesis^39^, hr-HPV and PAP test^38^.

Conventional Papanicolaou smears were collected following the standard procedure^40^. The test results were reported according to the Bethesda system^40^. Negative results for intraepithelial lesion or malignancy (NILM) were considered suitable inclusion criteria for the control group. Patients with the epithelial cell abnormalities or hr-HPV positive test were referred for further evaluation at tertiary hospitals with oncogynecology departments. For the detection of hr-HPV the pellet was used for the extraction of DNA with GeneJet Genomic DNA Purification Kit (Thermo Fisher Scientific, Vilnius, Lithuania) according to the manufacturer’s protocol and described elswhere^41^.

Colposcopies were performed by oncogynecologists specialized in cervical pathology (DV, JC, AS, DK, RPV) and biopsies were taken in accordance to International Research on Cancer guidelines that were supported by World Health Organization’s (WHO)^42^. The presence of the high-grade squamous intraepithelial lesion (HSIL), moderate (CINII) or severe (CINIII) dysplasia, as confirmed on cervical tissue from biopsies or conuses after loop electrosurgical excision procedure (LEEP), was an inclusion criterion into the research group. Patients with low-grade squamous intraepithelial lesion (LSIL, mild dysplasia CINI), who underwent LEEP procedure for reasons (for example long time > 2 year persistence) were assigned to the control group^43^. All histological evaluation of biopsies and conuses were performed and confirmed by two pathologists (ED and AC) at the National Cancer Institute and Kauno Klinikos, Lithuanian University of Health Sciences. A two-tier nomenclature, LSIL and HSIL, was used as a replacement for the former three-tiered “(-IN)” terminology, following the Waxman et al. recommendations^44^.

### Determination of Chlamydia trachomatis from cervical sampling

Cervical cells were collected from the cervical transformation zone by using a brush and were transformed to a vial of liquid preservative^40^ for the determination of *C. trachomatis* (CT). The deoxyribonucleic acid (DNA) extraction was performed for cervical swab samples using the GeneJET Genomic DNA Purification Kit (Thermo Fisher Scientific, Lithuania). The polymerase chain reaction (PCR) was performed using the primers CTP1 and CTP2 for the amplification of a 201-bp fragment of the cryptic chlamydial plasmid^45^. Primers were synthesized by the Metabion International AG, Germany. The primers used to generate a 201-bp fragment from the cryptic plasmid of *C. trachomatis* were CTP1 (5'-TAGTAACTGCCAClTCATCA-3') and CTP2 (5'-TTCCCCTTGTAATTCGTTGC-3').The PCR was performed with a final assay volume of 25 μl containing 2.5 μl 10x DreamTaq buffer (Thermo Fisher Scientific, Lithuania), 0.5 μl 10 mM deoxyribonucleotide triphosphate (dNTP), 1 μl of each primer (10 pmol/μl), 1 μl of the total genomic DNA isolated from clinical sample, 0.25 μL DreamTaq DNR polymerase (5U/μl), (Thermo Fisher Scientific, Lithuania) and 18.75 μl distilled water. Both positive and negative controls were performed in all PCR runs. The DNA extract from NATtrol™ CT/NG external run controls (ZeptoMetrix, USA) was used as a positive control. The PCR mix with primers and distilled water instead of DNA samples was used as negative control. The PCR amplification was performed in a programmable thermocycler Mastercycler Personal (Eppendorf, Germany) as follows: the DNA denaturation at 95 °C for 5 minutes, 40 cycles of denaturation at 95 °C for 30 s, annealing at 52 °C for 30 s, and elongation at 72 °C for 1 min, and a final elongation at 72 °C for 10 min. Finally, 10 μl amplified DNA were analyzed by electrophoresis on 1.5 % agarose gel and visualized by staining with ethidium bromide and illumination with ultraviolet light.

### Blood sampling

Written informed consent was obtained from every patient before the blood collection procedure. Peripheral blood samples were withdrawn from patients after NILM results obtained from conventional PAP smear. Venous blood was collected before conization procedure for HSIL and LSIL patients. The samples were collected into ethylenediamine tetraacetic acid (EDTA) containing tubes (Vacutainer, 367525, BD Biosciences, USA), preserved in +4 °C and used immediately for centrifugation at 2000g for 20 min to collect plasma, then aliquoted and stored at  80 °C until further analysis. Subsequent blood samples from every patient were collected six weeks and six months after conization following the same procedure.

### Determination of s-CAIX concentration in plasma

The plasma s-CAIX level was measured using the DIANA^©^ ligand-antibody-based method (performed at the Institute of Organic Chemistry and Biochemistry, Prague)^43^. For this method, the s-CAIX was captured by an antibody that was immobilized. The s-CAIX was probed with a small-molecule inhibitor that was attached to DNA and detected using the quantitative PCR^43^. The s-CAIX detection limit was 0.14 ± 0.04 pg/ml and the dynamic range was from 0.5 to 15,000 pg/ml.

### Statistical analysis

The statistical analysis package SPSS 26 was used for the data analysis. Results were presented in proportions, means, medians and ranges. The Mann Whitney U (MW-U) and Kruskall-Wallis tests (KWT) were used for non-parametric continuous data comparison of two (MW-U) or more than two (KWT) independent samples with the significance level less than 0.05. The Spearman’s correlation coefficient (r_s_) and Kendall’s tau_b (τ) was reported for correlation analysis. The odds ratio (OR) was reported with the confidence intervals CI at 95% significance level. All reposted *p* values are 2-sided. The standard deviations (SD) were calculated for 95% confidence level. Data were analyzed using SPSS 26 software.

### Ethical approval

The study was conducted according to the guidelines of the Declaration of Helsinki. The study was approved by the Lithuanian Bioethics committee (permission number 2016-03-22 Nr.: L-16–02/1) and Lithuanian Personal Data Protection Agency.

### Informed consent

Written informed consent was obtained from all subjects involved in the study. All subjects received information about study and voluntary signed a document approved by Lithuanian Bioethics committee and Lithuanian Personal Data Protection Agency.

## Results

### Clinicopathological characteristics of patient cohort

Table 1 summarizes patient’s sociodemographic characteristics. In 150 patients 77 were included to control group—73 with NILM and 4 with LSIL. In research group, 66 patients of HSIL and 6 patients of cervical cancer were included. One patient was excluded due to inappropriate histology report. The age of the patients ranged from 23 to 60 years (median 38.5 for control group and 36.5 for research group). There were no differences between the groups for partnership, contraception choice during lifetime and at the time of the study, menarche, coitarche, abortion and deliveries. In research group women had more partners and pregnancies.

**Table 1:** Demographic and clinical characteristics of the patients in research and control groups.

Value	Control group, median (percent or range)	Research group, median (percent or range)	*p* values
Age n = 142	38.5 (23–60)	36.5 (25–59)	0.28
Menarche n = 141	13 (11–18)	14 (11–19)	0.88
Coitarche n = 136	18 (10–26)	18 (13–25)	0.23
Number of partners n = 147	2 (1–5)	3 (1–10)	0.002*
Pregnancies n = 147	2 (0–6)	2 (0–10)	0.03*
Deliveries n = 147	2 (0–4)	1 (0–6)	0.16
Abortion n = 147	0 (0–3)	0 (0–5)	0.17
**Partnership N = 148**			
Married n = 107	58 (54.2%)	49 (45.8%)	> 0.05
Common-law partners n = 25	9 (36.0%)	16 (64.0%)	> 0.05
Divorced n = 3	3 (100.0%)	0 (0.0%)	> 0.05
Single n = 11	5 (45.5%)	6 (54.5%)	> 0.05
Widow n = 2	2 (100.0%)	0 (0.0%)	> 0.05
**Pelvic disease N = 148**			
Pelvic inflammatory disease positive n = 15	8 (53.3%)	7 (46.7%)	0.29
Endometriosis positive n = 5	4 (80%)	1 (20%)	0.20
*C. trachomatis* infection positive n = 11	5 (45.5%)	6 (54.5%)	> 0.05
**Contraception choice during the study**			
Barrier contraception n = 31	13 (41.9%)	18 (58.1%)	0.24
Chemical local contraception n = 1	0 (0%)	1 (100%)	0.49
Copper intrauterine device (IUD) n = 8	7 (87.5%)	1 (12.5%)	0.06
Hormonal contraception, except IUD n = 18	10 (55.6%)	8 (44.4%)	0.70
IUD with levonorgestrel n = 5	2 (40.0%)	3 (60.0%)	0.67
Lactation amenorrhea n = 1	1 (100%)	0 (0%)	0.09
Coitus interruptus n = 7	6 (85.7%)	1 (14.3%)	0.09
Total who used any contraception n = 71	39 (54.9%)	32 (45.1%)	0.40
Total who did not use contraception n = 79	38 (48.1%)	41 (51.9%)	0.40
Total in the group n = 150	77 (51.3%)	73 (48.7%)	0.40
**Contraception choice during the life**			
Barrier contraception n = 100	57 (57.0%)	43 (43.0%)	0.06
Chemical local contraception n = 7	3 (42.9%)	4 (57.1%)	0.71
Copper intrauterine device (IUD) n = 28	17 (60.7%)	11 (39.3%)	0.29
Hormonal contraception, except IUD n = 79	45 (57.0%)	34 (43.0%)	0.17
IUD with levonorgestrel n = 13	7 (53.8%)	6 (46.2%)	1.0
Coitus interruptus n = 2	2 (100%)	0 (0%)	1.0
Patients, who used any contraception n = 130	70 (53.8%)	60 (46.2%)	0.11
Patients, who did not use contraception n = 20	7 (35.0%)	13 (51.9%)	0.11
Total in the group n = 150	77 (51.3%)	73 (48.7%)	0.11

*—statistically significant difference. Values are medians with inter-quartile ranges for continuous data and frequency (percentage, %) for categorical data. The listed p values of statistical tests were calculated using Mann–Whitney U test for continuous data and the χ2 or Fisher’s exact test for categorical data. Numbers slightly vary within groups due to missing answers.

These differences are related to the risk factors since number of contacts increases the probability for hr-HPV acquisition, the main risk factor for the development of intracervical neoplasia.

Women were asked about non-communicable diseases, but this was not a criterion for inclusion into the study. Most of patients (n=102; 75.0%) of respondents reported to be free from other diseases. More than half 54 patients (77.6%) without reported disease were in the intracervical neoplasia (HSIL) group and 48 (47.1%) women in the control group (*p*<0.01). Four women had hypothyroid disease and all of them were from control group (*p*=0.054). Primary arterial hypertension was prevalent in both groups—7 patients from control group and 4 patients from research group reported the disease (*p*=0.3). Asthma was diagnosed for 1 patient in control group and 3 patients in research group. Anemia—for two women in control group. One woman had depression in research group and one woman reported dermatitis in control group.

### Association of carbonic anhydrase IX (CAIX) concentration in blood plasma and contraception choice

In this study we compared the mean s-CAIX concentration between the control and research group in general and in each contraception choice group. The aim was to find out if the contraception choice is associated with s-CAIX level. The mean concentration of s-CAIX was 75.0 pg/ml, with maximum value 459.20 pg/ml. After comparison of mean s-CAIX level in plasma in the control group (80.6 pg/ml) and in research group (68.9 pg/ml), no statistically significant difference was detected (*p*=0.17).

Contraceptive choice was not associated with s-CAIX level before conization (MW-U *p*>0.05) and 6 weeks after surgery (MW-U *p*>0.05). This change of the concentration of s-CAIX did not differ between various contraceptive choices in HSIL and in control groups (WSR *p*>0.05).

### Association of carbonic anhydrase IX (CAIX) concentration in blood plasma and age

**Figure 1:**
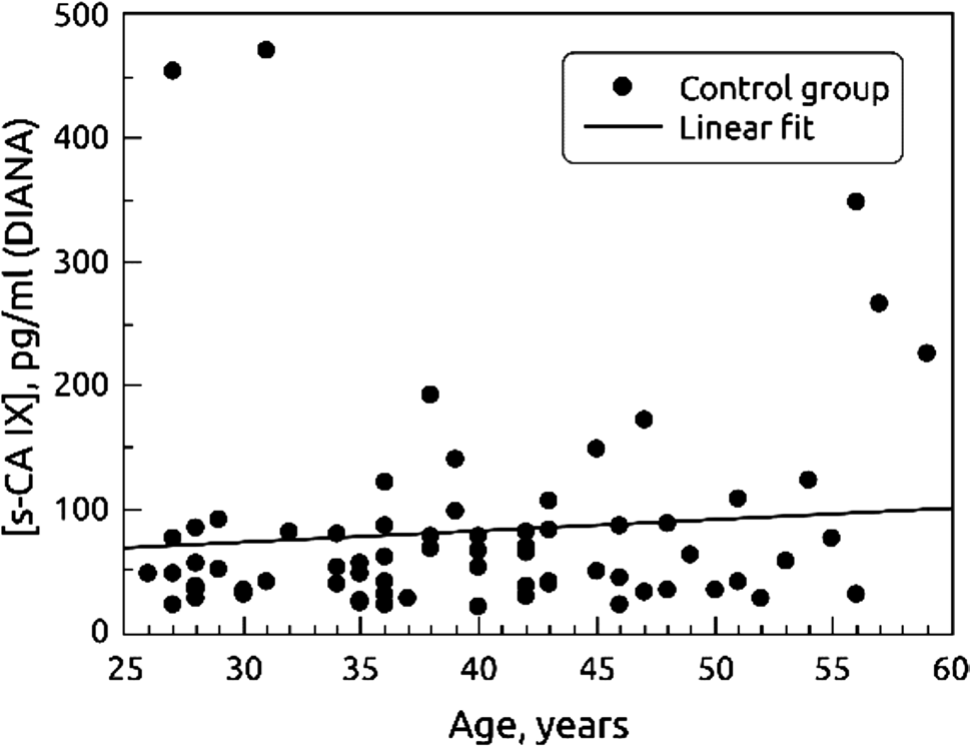
Association between the age and mean s-CAIX concentration in blood plasma among 75 women from control group (rs = 0,13, *p* = 0.27).

**Figure 2:**
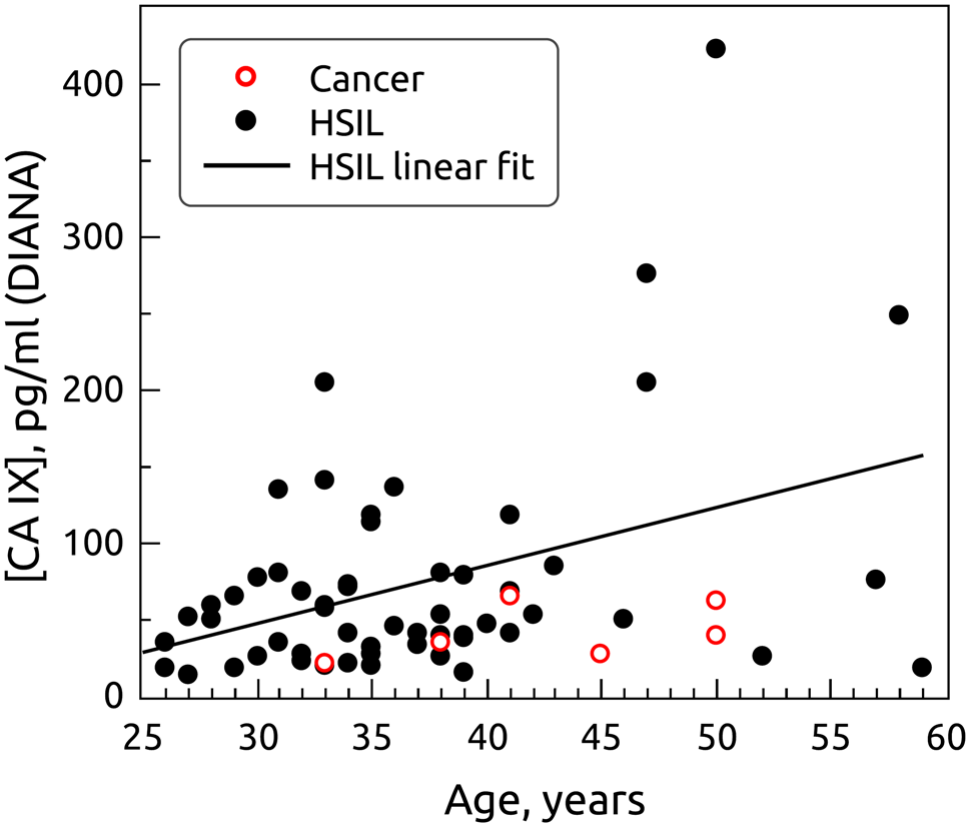
Association between the age and mean s-CAIX concentration in blood plasma among 56 women with HSIL (rs = 0,27, *p* = 0.04).

The s-CAIX concentration correlated with age – we detected higher level of s-CAIX among older women (rs=0.19, *p*=0.03). In the group of women without cervical abnormalities, correlation between s-CAIX level and age was insignificant (rs=0.13, *p*=0.27) (Fig 1). However, in women with HSIL but without cancer, correlation between s-CAIX level and age was stronger (rs=0.27, *p*=0.04) (Fig. 2). In cancer group the correlation was not significant, however only 6 cases of cancer were in the sample (τ =0.41, *p*=0.25).

### Association of carbonic anhydrase IX (CAIX) concentration in blood plasma and pelvic inflammation

Pelvic inflammatory disease was another indication associated with the elevated s-CAIX concentration in patient plasma. Among 15 women with pelvic disease, 11 had diagnosis of *C. trachomatis* (N=6 in HSIL and 5 in control groups) and 5—endometriosis. One woman from control group was diagnosed with both—endometriosis and *C. trachomatis* infection.

Firstly, we wanted to test if pelvic inflammation or *C. trachomatis* infection was independent risk factor for elevated s-CAIX in women. Mean age of women suffering from pelvic inflammation was 40.70 years (SEM=2.3) and 38.10 years (SEM=0.76) for women, without pelvic inflammation (MW-U *p*=0.78) in total sample. The mean age was similar in women with and without pelvic inflammation in HSIL group (MW-U *p*>0.05). There was no difference in prevalence of pelvic inflammation or *C. trachomatis* between control and HSIL group (MW-U *p*>0.05).

In total sample the concentration of s-CAIX was elevated in women with pelvic disease. Mean s-CAIX level was 100.62 pg/ml (SEM=16.40) for patients with pelvic inflammation versus 71.19 pg/ml (SEM=6.99) for women without the pelvic disease (MW-U *p* = 0.01).

After comparison of the s-CAIX level and *C. trachomatis* we did not find correlation in total sample. Patient with *C. trachomatis* did not have elevated s-CAIX concentration in plasma (mean s-CAIX concentration was 76.96 pg/ml among *C. trachomatis*-positive women and 118.14 pg/ml among *C. trachomatis*-negative women; MW-U *p*=0.965). When we analyzed association of s-CAIX level and *C. trachomatis* in women suffered from pelvic inflammation, no statistically significant result was found. Among women with pelvic inflammation, the mean s-CAIX level did not differed between the *C. trachomatis* positive and negative women (93.11 pg/ml versus 108.31 pg/ml; MW-U *p*=0.096).

**Figure 3:**
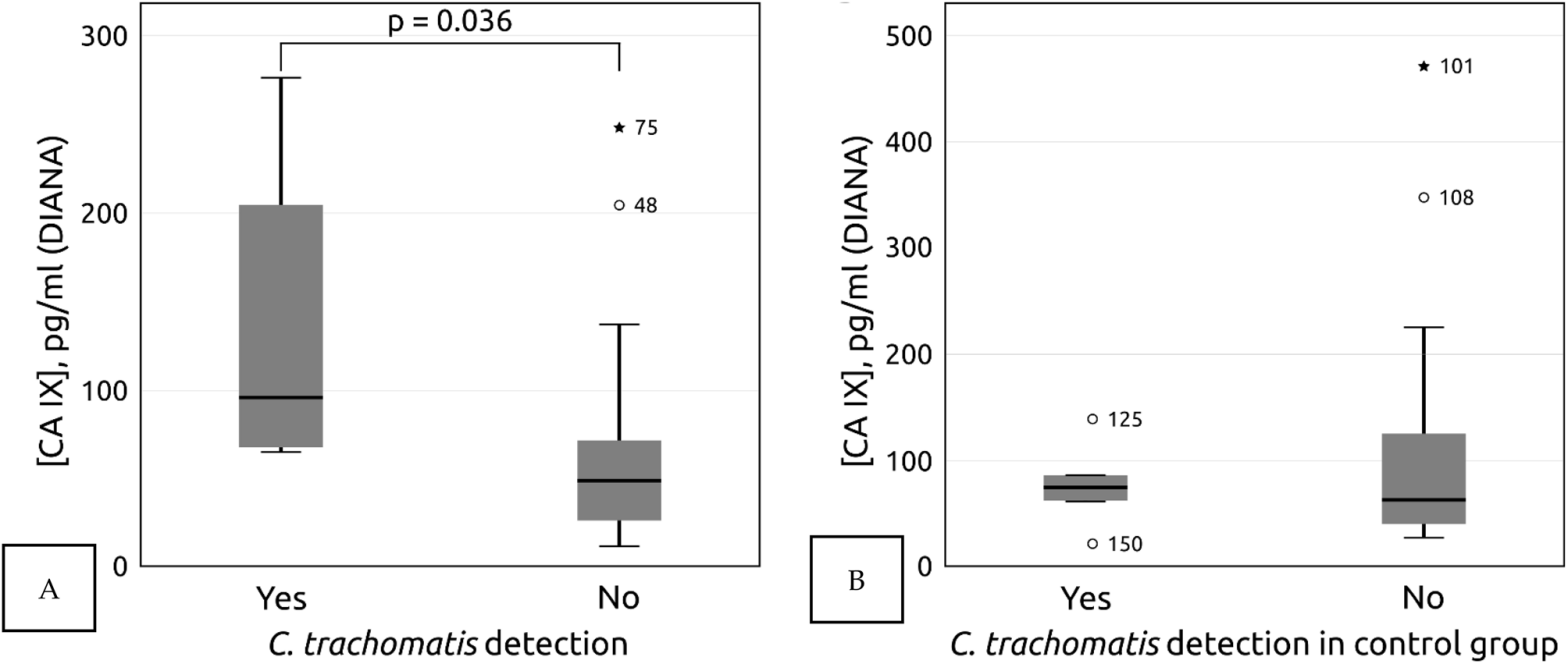
(**A**) Difference of mean s-CAIX level among ≥ HSIL patients (on the left *C. trachomatis* positive N = 6, on the right and *C. trachomatis* negative women N = 18). (**B**) Difference of mean s-CAIX level among controls (on the left *C. trachomatis* positive N = 5, on the right *C. trachomatis* negative N = 15).

We analyzed the correlation of the concentration of s-CAIX in plasma and the presence of pelvic disease among HSIL patients. In HSIL group, women with inflammatory pelvic disease had statistically significant increase of mean s-CAIX concentration (117.83 pg/ml (SEM = 34.30) compared with women without pelvic inflammation (mean 63.20 pg/ml; SEM = 8.35; MW-U *p *= 0.047). In the HSIL group, s-CAIX concentration in blood plasma was higher for women who suffered from *C. trachomatis* infection (134.43 pg/ml (SEM = 35.52) than for those who did not (70.52 pg/ml (SEM = 15.63); MW-U *p* = 0.028 (Fig. 3).

This tendency was not observed in the control group (mean 90.58 pg/ml (SEM=17.23) for inflammatory pelvic disease patient versus 78.78 pg/ml (SEM=11.27) for inflammatory disease-free women; MW-U *p* = 0.131) or for *C. trachomatis* (MW-U *p *> 0.05 (Fig. 3).

We could not determine if endometriosis without *C. trachomatis* was associated with the elevated CAIX level because nobody reported endometriosis in the HSIL group. Among women in the control group without *C. trachomatis* infection, only two reported endometriosis.

### Association of carbonic anhydrase IX (CAIX) concentration in blood plasma and non-communicable diseases

The prevalence of non-communicable diseases such as hypertension, diabetes mellites and others is related to age. The weak correlation of age and non-communicable disease was observed in our study (τ = 0.239, *p *= 0.01). These reported diseases were not associated with higher s-CAIX level in plasma, both for all women (MW-U *p* = 0.2), as well as for HSIL group (MW-U *p* = 0.27).

The mean s-CAIX concentration was similar among women free from any non-communicable disease and ones who suffered from endometriosis in total sample (MW-U *p* = 0.98) and in HSIL group (*p *= 0.2). The same result was observed comparing healthy women with ones suffering from the primary arterial hypertension (*N* = 4 in HSIL group and N = 7 in control group), both in total sample (MW-U *p* = 0.6) and in HSIL group (MW-U *p* = 0.3). Similarly, the hypothyroid disease (N = 4 in control group) was not associated with elevated s-CAIX level in total sample (MW-U *p* = 0.37). No one reported thyroid disease in HSIL group.

### Association of s-CAIX concentration in plasma and smoking

One of the factors, increasing hypoxia in human body is smoking. The concentration of s- CAIX did not differ between smokers and non-smokers (MW-U *p* = 0.58). More women reported smoking in HSIL group than in control (68.4% (*N* = 39) versus 31.6% (N = 18), χ2 16.15 df = 1; *p* < 0.01). Risk to have HSIL for smokers was higher (OR = 0.24, CI95% 0.12-0.49). The proportion of every day smokers was similar between control (18.2% N = 14) and HSIL (24.3% N = 17) groups, but more people reported occasional smoking in HSIL (30.0 % N = 21) than in control group (5.2% N = 4; *p*<0.001). Non-smokers made 76.6% (N = 59) in the control group versus 45.7% (N = 32) in HSIL group (*p*<0.001).

The concentration of s-CAIX was similar for heavy smokers, occasional smokers, and non-smokers during first (KWT p = 0.28), second (KWT p = 0.84) and third (KWT p = 0.68) blood sampling. Among heavy smokers the concentration was higher in older women (rs = 0.52, p = 0.005). We did not find this association among occasional (rs =  0.12 p = 0.61) or non-smokers (rs = 0.16 p = 0.14). The association of s-CAIX level and age was even stronger in heavy smokers diagnosed with HSIL (rs = 0.68, p = 0.008).

The concentration of s-CAIX was not related to age in heavy-smokers controls (τ = 0.18 p=0.40), neither in occasional smokers HSIL (τ =  0.02 *p* = 0.93), nor in non-smokers HSIL (τ=0.12 p=0.38).

## Discussion

Association of s-CAIX level with the risk factors for HSIL progression were analyzed in this study. As CAIX participates in pH regulation^22^, hypoxia is associated with the elevation of CAIX expression and with cancer^46^. Inhibition of CAIX is under research as a therapeutic drug target^22,23^.

The elevated concentration of s-CAIX in plasma, a systemic hypoxic marker, was associated with the major risk factors—the age, *C. trachomatis* infection and heavy smoking in HSIL, but not control group. These factors are known as contributing to the progression of intracervical neoplasia towards cancer^11,39,47^. These findings are important for identification of patient stratification markers within HSIL and urge for further observatory translational research analyzing HSIL progression prognosis and an elevated s-CAIX in patients.

In our previous study, we analyzed the association of plasma s-CAIX level with a high-grade intracervical neoplasia^43^. We found that s-CAIX could be detected in patients’ plasma. However, s-CAIX concentration could not help to discriminate between the HSIL and the control groups^43^. These data are in line with the findings of other researchers. The level of s-CAIX in plasma did not help to discriminate the locally advanced breast cancer from healthy controls (*p* = 0.07) but was significantly elevated in the metastatic breast cancer (*p* < 0.001)^48^ and non-small cell lung cancer^35^.

However, our study showed some differences in s-CAIX plasma level. The concentration of s-CAIX correlated with the age and pelvic disease, especially *C. trachomatis* infection in HSIL group, but not in the control group. The explanation of correlation with pelvic disease could be the fact that CAIX is detected in mesothelial cells (fluids). Similar findings have been described by Ananthanarayanan^49^, and Capkova et al^50^. Mesothelium, both reactive and malignant were positive for CAIX immunostaining. The *C. trachomatis* infection upregulates molecular biomarkers podoplanin, Wilms’ tumour gene 1, osteopontin and inflammatory cytokines in human mesothelial cells^51^. Furthermore, there was an association between cervical cancer, especially squamous cell adenocarcinoma, and chronic *C. trachomatis* infection in hr-HPV infected individuals^52,53^.

*C. trachomatis* causes permanent cytological changes in host mesothelial cells—increases cell proliferation, resistance to apoptosis, enforces survival and predispose them to transformation^51^. It seems that *C. trachomatis* contributes to oncogenic transformation of hr-HPV infected cells, thus leading from low grade to high grade intracervical neoplasia. *C. trachomatis* induces inflammation that leads to pro-angiogenic (VEGF) and pro-metastatic (IL-6, IL-8 and TNF-α) cytokine overexpression^51^. IL-6 through STA3 axis promotes activation of the transcription of HIF-1 (hypoxia induced factor–1) which activates CAIX gene and consequently increases the CAIX protein production^54^.

During the *C. trachomatis* infection in lower pelvis, free fluid could be observed, which could be reabsorbed by the surrounding tissue. The CAIX is produced in mesothelial cells and could be reabsorbed into blood steam. However, why we detected this elevation only in HSIL, but not in the control group, remains unclear. Excision of the lesion did not lead to a dropped level of s-CAIX. However, we did not evaluate the outcome or the *C. trachomatis* status and lower pelvic inflammation after conization. The cohort study with close evaluation of inflammation markers in lower pelvis could help to clarify if s-CAIX level is associated to chronic inflammation in lower pelvis or remains additional marker for HSIL progression/recurrence.

Only few researchers reported the association of CAIX with human age in cancer patients. In brain astrocytoma study, the CAIX cellular immunostaining was increasing with higher patient’s age^55^. However, the CAIX immunostaining was an independent prognostic factor from patient’s age, revealing importance of CAIX both in tumorigenesis and aging process. This trend was not observed in head and neck squamous cell carcinoma^56^, tongue squamous cell carcinoma^57^, and pancreatic cancer^58^ cases, but the range of age groups was not so wide as in astrocytoma study.

There is a lack of research about s-CAIX level in plasma and its relation to normal aging. However, CAIX knock-out mice had vacuolar degenerative changes in their brains which were related to abnormal locomotor activity and poor performance in memory test at eight to ten months age^59^. This means that aging contributes to changes, associated with the lack of CAIX. Furthermore, CAIX knock-out mice also had presented a hyperplasia of stomach mucosa, impaired pH regulation, and chronic inflammation^46^. The authors discuss that the aging-associated decrease of blood flow in stomach mucosa is compensated by CAIX expression to decrease acidosis in hypoxia^46^.

During physiological aging, hypoxia level increases in human body and causes age-rated damage^60^. However, additional factor, such as intermittent hypoxia could aggravate age-related process. For example, intermitted hypoxia induces several molecular, biochemical, and cellular changes which facilitates a better environment for tumor cell growth^61^. Systemic intermittent hypoxia aggravates this damage through hypoxia induces factor alfa (HIF-1α) and sirtuin (silent mating type information regulation 2 homolog) 1 (SIRT1) pathways^60^. Several researchers showed that HIF-1α is overexpressed in HSIL and cervical cancer lesions^62^. HIF-1α expression with hr-HPV infection could contribute toward precancerous lesion (HSIL) progression into tumor^63^. Also, it is known that SIRT1 overexpression contributes to epigenetic mechanism of hr-HPV infected cells to maintain undifferentiated stage – essential for transformation of normal cervical epithelium to HSIL lesion^64^. These data evidence that systemic intermittent hypoxia could contribute to hr-HPV infection effect on host cells inducing preneoplastic changes. The level and exposure of intermittent hypoxia may differ among the HSIL patients and higher level of s-CAIX could be a marker for advanced hypoxia process. Thus, together with other risk factors, this process in HSIL group could contribute too poorer prognosis.

The data, analyzing association between smoking and CAIX expression is contradictory. There was no association of the CAIX level in tumor tissues with smoking status in patients with non-small lung cancer^35^. The methylation and expression of CAIX gene profile was not modified in chronic smokers in oral cancer tissue as well^65^. However, other researchers found that HIF-1α and hypoxia is important for therapeutic response in outcomes of nicotine users suffering from cancer^66^. High expression of CAIX was found in resected pulmonary metastases and corresponding primary tumors that was a risk factor for early spreading to the lung (*p* < 0.001)^67^. The expression of CAIX in this study was affected by the smoking habits^67^. Same researcher treated HT29 cells with nicotine and found an induction of CAIX in vitro^67^. This induction was associated with STAT3 phosphorylation and was independent of HIF-1α^67^. The research design could be the reason for these discrepancies in conclusions. The effects of nicotine on HIF-1a expression were transient, returning to baseline levels within 2-3 days after nicotine removal^66^. We analyzed heavy smokers that currently were using nicotine and only in this group the s-CAIX level was increased in older respondents. As age and heavy smoking are independent poor prognosis factors for intracervical neoplasia, elevated s-CAIX could be a marker of higher risk group among those women and contribute for patient stratification.

We found a correlation of s-CAIX level in plasma and older age in HSIL group which was not associated to pelvic inflammation. We have not analyzed systemic intermitted hypoxia in women and have no data on HSIL recurrence in patients with HSIL and elevated s-CAIX concentration. However, our research puts inside that the systemic mechanisms are involved in HSIL transformation, and they are not modified after excision of lesion. This could explain why we did not find any correlation between s-CAIX blood plasma level and CAIX immunohistochemical staining in conuses^43^. Also, despite the fact, that the control group had higher proportion of non-communicable disease, we did not notice any correlation with age and these diseases. Based on our findings, further research is essential for clarification of systemic factors, predisposing pre-carcinogenesis and for development of preventative strategies for cervical cancer elimination.

Limitation of this study is the lack of long term follow up of patients with HSIL and elevated s-CAIX. Further research is necessary for better understanding of s-CAIX measurement for prognosis. However, the recurrence of the disease could occur during several decades suggesting a necessity for longitudinal observatory study.

## Conclusions

We have examined potential players in HSIL patient stratification. The level of s-CAIX was found to be elevated in a part of patients with known risk factors for HSIL progression—advanced age, heavy smoking, and *C. trachomatis* infection. The data suggest that hypoxia markers, one of them s-CAIX, may have a patient stratification utility in the decision making for women discussing the risk of treatment postponement.

## Data Availability

The data that support the findings of this study are available from Vilnius University Life Science Center Institute of Biotechnology, Department of Biothermodynamics and Drug Design, but restrictions apply to the availability of these data, which were used under license for the current study, and so are not publicly available. Data are however available from the authors upon reasonable request and with permission of Vilnius University Life Science Center Institute of Biotechnology, Department of Biothermodynamics and Drug Design.
